# Tachycardia in a teenager

**DOI:** 10.1007/s12471-021-01610-6

**Published:** 2021-08-03

**Authors:** Y. R. Persia-Paulino, J. Rozado, D. Perez

**Affiliations:** 1 Cardiology Department, Central University Hospital of Asturias, Oviedo, Spain; 2 Electrophysiology Department, Central University Hospital of Asturias, Oviedo, Spain

A 14-year-old patient was brought to the emergency room (ER) due to palpitations. Her father had suffered sudden death aged 30 years. She had no toxic habits. When examined 4 months previously due to palpitations, her electrocardiogram (ECG) and echocardiogram were normal. On arrival at the ER she was conscious, with a blood pressure of 96/64 mm Hg, heart rate of 160 beats/min and a ventilation frequency of 14 breaths/min. Physical examination findings were severe tachycardia and normal heart sounds with no murmurs, as well as well-ventilated lung zones with no additional sounds. Fig. 1 shows the ECG at ER admission.

**Fig. 1:**
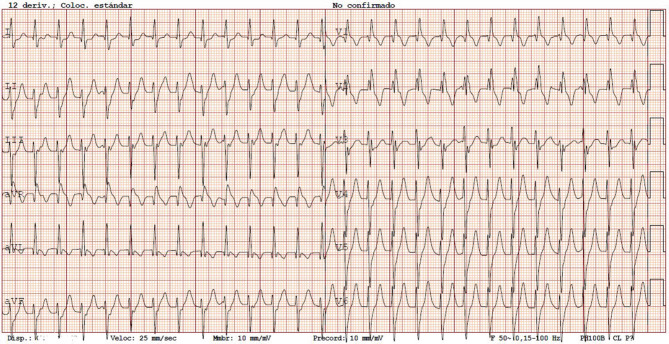
The 12-lead electrocardiogram on admission to the emergency room

Vagal and modified vagal manoeuvres were performed but had no effect on the tachycardia. Three doses of intravenous adenosine (6 mg, 12 mg and 12 mg) were administered but neither stopped the tachycardia nor lowered the heart rate. During continuous ECG monitoring at the ER, her heart rate increased to 210 beats/min when standing up, returning to 166 beats/min at rest. Electrical cardioversion was performed due to hypotension (80/60 mm Hg) after adenosine administration; the ECG is shown in Fig. 2.

**Fig. 2:**
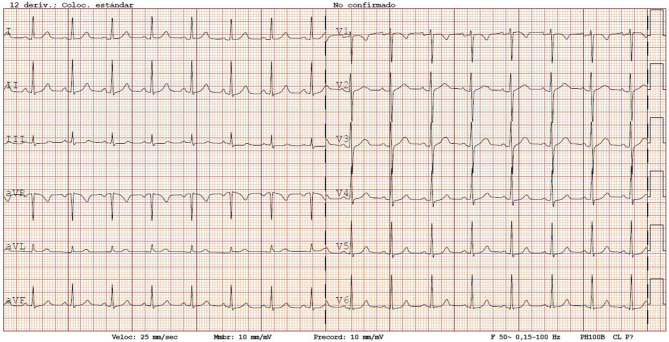
The 12-lead electrocardiogram after direct current cardioversion due to hypotension

What do you think was the cause of this tachycardia?

## Answer

You will find the answer elsewhere in this issue.

